# Subcellular localization and trafficking of phytolongins (non-SNARE longins) in the plant secretory pathway

**DOI:** 10.1093/jxb/erw094

**Published:** 2016-03-09

**Authors:** Carine de Marcos Lousa, Eric Soubeyrand, Paolo Bolognese, Valerie Wattelet-Boyer, Guillaume Bouyssou, Claireline Marais, Yohann Boutté, Francesco Filippini, Patrick Moreau

**Affiliations:** ^1^Centre for Plant Sciences, Faculty of Biological Sciences, University of Leeds, Leeds LS2 9JT, UK; ^2^Faculty of Clinical and Applied Sciences, School of Biomedical Sciences, Leeds Beckett University, Portland Building 611, Leeds Beckett University City Campus, LS1 3HE, Leeds, UK; ^3^CNRS-University of Bordeaux, UMR 5200 Membrane Biogenesis Laboratory, INRA Bordeaux Aquitaine, 71 Avenue Edouard Bourlaux, CS 20032, 33140 Villenave d’Ornon, France; ^4^Molecular Biology and Bioinformatics Unit, Department of Biology, University of Padova, Viale G. Colombo 3, 35131 Padova, Italy; ^5^Bordeaux Imaging Center, UMS 3420 CNRS, US004 INSERM, University of Bordeaux, 33000 Bordeaux, France

**Keywords:** ER export YF motif, longin domain, phytolongins, protein targeting, secretory pathway, subcellular localization.

## Abstract

SNARE proteins are central elements of the machinery involved in membrane fusion of eukaryotic cells. In animals and plants, SNAREs have diversified to sustain a variety of specific functions. In animals, R-SNARE proteins called brevins have diversified; in contrast, in plants, the R-SNARE proteins named longins have diversified. Recently, a new subfamily of four longins named ‘phytolongins’ (Phyl) was discovered. One intriguing aspect of Phyl proteins is the lack of the typical SNARE motif, which is replaced by another domain termed the ‘Phyl domain’. Phytolongins have a rather ubiquitous tissue expression in Arabidopsis but still await intracellular characterization. In this study, we found that the four phytolongins are distributed along the secretory pathway. While Phyl2.1 and Phyl2.2 are strictly located at the endoplasmic reticulum network, Phyl1.2 associates with the Golgi bodies, and Phyl1.1 locates mainly at the plasma membrane and partially in the Golgi bodies and post-Golgi compartments. Our results show that export of Phyl1.1 from the endoplasmic reticulum depends on the GTPase Sar1, the Sar1 guanine nucleotide exchange factor Sec12, and the SNAREs Sec22 and Memb11. In addition, we have identified the Y48F49 motif as being critical for the exit of Phyl1.1 from the endoplasmic reticulum. Our results provide the first characterization of the subcellular localization of the phytolongins, and we discuss their potential role in regulating the secretory pathway.

## Highlight

The subcellular localization and trafficking in the plant secretory pathway of four proteins from a new subfamily of non-SNARE longins, called phytolongins, has been established.

## Introduction

SNAREs are small proteins that are anchored to membranes through a carboxy (C)-terminal transmembrane α-helix. SNAREs are classified as Q- (also termed t-) or R- (also termed v-) depending on which amino acid [glutamine (Q) or arginine (R)] is located at the central position of the core SNARE motif. Q-SNAREs are further subdivided into Qa-, Qb- and Qc-SNAREs. It is assumed that a SNARE complex results from the assembly of one copy of each of the Qa-, Qb-, and Qc-SNAREs on the acceptor membrane with an R-SNARE on the donor membrane. The SNARE complex formed will dock vesicles with their targeted membranes and allow lipid bilayer fusion. Most SNAREs contain in their amino (N)-terminal part an evolutionarily conserved SNARE motif of approximatively 60 residues. Additional domains in this N-terminus part can fold on to the SNARE motif to regulate fusion by hiding (closed conformation) or allowing (open conformation) the SNARE motif to assemble into the SNARE complex.

Despite the high evolutionary conservation of SNAREs across biological kingdoms, there is an intriguing diversification of SNAREs to meet specific requirements and adaptations in animals and plants. This diversification is particularly obvious within the R-SNAREs subclass. Brevins, which are characterized by a short, non-conserved N-terminal domain, are present only in Opisthokonta (animals, fungi, and yeasts), and their number is increased in animals due to the special role that synaptobrevins play in synaptic transport in neurons. In contrast, plants have diversified R-SNAREs termed longins, named for their long domain, as opposed to the short brevins. Longins are conserved in, and essential to, all eukaryotes (Rossi *et al.*, 2004) and are differentiated by the presence of an N-terminal longin domain (LD) in addition to the SNARE motif. In particular, although the longin subclass of VAMP7 genes is found in only one copy in animals, 11 isoforms are present in *Arabidopsis thaliana*. Homology-based classification allowed the identification of two branches in plant VAMP7 evolution (VAMP71 and VAMP72). Therefore, in the flowering plant model *A. thaliana*, the 11 VAMP7 proteins have been grouped into two subclasses, VAMP711–VAMP714 and VAMP721–VAMP727 (Lipka *et al.*, 2007; Sanderfoot, 2007).

In recent years, parallel investigations on animal and plant VAMP7 have shown conserved and special features. The LD has been shown to play a crucial role in determining subcellular localization of VAMP7 proteins in both animals (Martinez-Arca *et al.*, 2003) and plants (Uemura *et al.*, 2005). However, intracellular patterns differ, with animal VAMP7 being mainly endosomal (Chaineau *et al.*, 2009) whereas plant VAMP7 proteins are found in several compartments [endoplasmic reticulum (ER), Golgi, endosomes, vacuole, and plasma membrane (PM); Uemura *et al.*, 2004]. The characterization of some plant VAMP7 proteins has led to the unveiling of special molecular features that play a role in their involvement in plant physiology and immunity (Ebine *et al.*, 2008; Kwon *et al.*, 2008; Leshem *et al.*, 2010; Levine *et al.*, 2001).

In VAMP7 (as well as in the longins Sec22b and Ykt6), the LD is known to bind intramolecularly to the SNARE motif to mediate closed conformation, hence regulating membrane fusion and subcellular sorting (Mancias and Goldberg, 2007; Tochio *et al.*, 2001; Vivona *et al.*, 2010). It can also interact with other proteins and protein domains, such as the δ subunit of the adaptor protein complex AP-3 (Martinez-Arca *et al.*, 2003), the SNARE-like region of Hrb (Pryor *et al.*, 2008), and the Varp protein (Burgo *et al.*, 2009). Evidence that the LD is not just a ‘SNARE regulatory domain’, as originally proposed, is further confirmed by (i) conservation of the LD fold in non-SNARE trafficking proteins such as the σ and μ subunits of the AP-2 complex and the SRx domain within the signal recognition particle complex, and by (ii) the existence of non-SNARE gene isoforms (Sec22a and Sec22c) and splice variants (TI-VAMP/VAMP7b) of longins (Rossi *et al.*, 2004; Schlenker *et al.*, 2006).

More strikingly, a subfamily of four non-SNARE longins has been discovered recently (Vedovato *et al.*, 2009); interestingly, they are highly conserved in, and specific to, land plants. These new proteins were named ‘phytolongins’ (Phyl) and given the abbreviated names Phyl1.1, Phyl1.2, Phyl2.1, and Phyl2.2. They and the classic longins from the VAMP7 family share their overall domain architecture, with a central region nested between the LD at the N-terminus and a transmembrane domain (TMD) at the C-terminus. However, in the phytolongins, the central SNARE motif of classic longins is absent and is replaced by a new domain called the ‘Phyl domain’, whose function is as yet unknown. Sequence homology and structural modelling of the LD indicate that the LD of phytolongins is closer to that of VAMP7 than of other longins. Additionally, an evolutionary analysis demonstrated that this non-SNARE longin subfamily emerged as a new branch from VAMP7 proteins in land plants and in turn gave rise to two subgroups of phytolongins, each of which has two members in *A. thaliana* (Phyl1.1/Phyl1.2 and Phyl2.1/Phyl2.2) (Vedovato *et al.*, 2009). Recent investigations have indicated that the longins represent a large superfamily that includes five major families—*sensu stricto* longins (both SNAREs and non-SNAREs, including phytolongins), adaptins, sedlins, SANDS, and targetins (De Franceschi *et al.*, 2014)—which are involved or potentially involved in mediating many important intracellular trafficking steps.

Phytolongins have a rather ubiquitous tissue expression, with the exception of Phyl2.2, which appears to be expressed only in juvenile and adult leaves and flowers (according to Genevestigator; https://genevestigator.com/gv/); these data highlight the possible general involvement of phytolongins in plant development. Moreover, recent proteome-wide analyses have shown the LD to be a very ancient domain and a building block of the subcellular trafficking machinery that is crucial to eukaryotic cell life and organization, as it is more highly conserved across organisms and spread among trafficking machineries than is the SNARE domain (De Franceschi *et al.*, 2014). Despite these indications, the phytolongins appear to be new family of longins for which intracellular characterization is yet to be performed. This prompted us, in a first approach, to determine their subcellular localization and potential targeting to the secretory pathway. Our results reveal that the four phytolongins are distributed along the secretory pathway, with Phyl2.1 and Phyl2.2 being localized at the ER network, Phyl1.2 associated with the Golgi bodies, and Phyl1.1 distributed between the Golgi bodies and the PM with a major localization at the PM. Moreover, we established that the differential intracellular localization between the ER-located Phyl2 proteins and Golgi/PM-located Phyl1 proteins relies on a Y48F49 motif contained within the LD.

## Materials and methods

We cloned the coding sequence of the genes for the four Arabidopsis phytolongins by PCR using their genomic DNA as a template, taking advantage of the single-exon structure shared by the genes. Using Invitrogen Gateway technology, each phytolongin coding sequence (native or mutated) was subsequently subcloned into a vector for expression of either green fluorescent protein (GFP)- or yellow fluorescent protein (YFP)-tagged fusion chimeras. The corresponding constructs were then sequenced and transformed into *Agrobacterium tumefaciens* (strain GV301) for subsequent analysis in tobacco leaf epidermal cells.

### Plant material and transient expression systems

Four-week-old tobacco (*Nicotiana tabacum* cv Xanthi) plants grown in a greenhouse at 22–24 °C were used for *A. tumefaciens*-mediated transient expression (Batoko *et al.*, 2000). *A. tumefaciens* carrying the constructs in the transforming binary vectors were cultured at 28 °C to stationary phase (approximately 24h), washed, and resuspended in infiltration medium [MES 50mM pH 5.6, glucose 0.5% (w/v), Na_3_PO_4_ 2mM, acetosyringone (Aldrich) 100mM from 200mM stock in dimethyl sulfoxide]. The bacterial suspension was inoculated into plants using a 1ml syringe without a needle by gentle pressure through a small puncture on the abaxial epidermal surface (Brandizzi *et al.*, 2002a, b). Transformed plants were then incubated under normal growth conditions for two days at 22–24 °C.

### Confocal microscopy and expression of the different constructs

Transformed leaves were analysed 48h after infection of the lower epidermis. Images were captured either with a Leica TCS SP2 confocal laser scanning microscope with a ×63 oil immersion objective (performed at the plant imaging facility of the Bordeaux Imaging Center, http://www.bic.u-bordeaux2.fr) or on a Zeiss LSM700 inverted confocal with a ×40 oil immersion objective (performed at at the Centre for Plant Sciences, University of Leeds) [488nm laser for GFP and YFP, 555nm laser for red fluorescent protein (RFP)]. For imaging cyan fluorescent protein (CFP), GFP, and YFP constructs, excitation lines of an argon ion laser of 458, 488, and 514nm, respectively, were used; for imaging RFP, the excitation line of a He/Ne ion laser of 543nm was used, and line switching using the multi-track facilities of the microscopes was used for the alternative acquisitions. Imaging settings were as described by Brandizzi *et al.* (2002a, b).

The vectors used for the expression of the GTP-blocked and GDP-blocked mutant forms of Sar1 were purchased from Professor F. Brandizzi (Plant Research Laboratory, Michigan State University, USA). Besides the invisible *Sar1* genes, these double vectors also carry an SKL-CFP peroxisomal marker used as a reporter to monitor cells expressing the Sar1 mutants. Similarly, ST-RFP was used as a reporter in double vectors over-expressing *Sec12* mutants, according to Gershlick *et al.* (2014). The GFP-Rem1.3 construct was provided by S. Mongrand (UMR 5200; Perraki *et al.*, 2012; Raffaele *et al.*, 2009). For the RFP-SYP121 construct, the RFP gene was subcloned in place of YFP in YFP-SYP121 (Bottanelli *et al.*, 2012), using the same restriction enzymes. The other vectors for RFP-CBL6, RFP-SYP61, RFP-VSR2, and RFP-Rha1 were kindly provided by Professor J. Denecke (Centre for Plant Sciences, University of Leeds, UK). Quantification of co-localizations in some experiments was done according to Gershlick *et al.* (2014).

### Detergent-insoluble membrane fraction isolation and western blot analyses

GFP-Phyl1.1-transformed leaves of 4-week-old tobacco (*N. tabacum* cv Xanthi) greenhouse-grown plants were collected and homogenized on ice in the presence of 10mM KH_2_PO_4_ (pH 8.2) with 0.5M sorbitol, 5% (w/v) PVP40, 0.5% (w/v) BSA, 2mM salicylhydroxamic acid, and 1mM PMSF. After filtration, the homogenate was subjected to successive centrifugations at 1000 *g* for 10min, 10000g for 10min, and 150000g for 60min. The resulting microsomal pellet was suspended in 10mM KH_2_PO_4_ (pH 8.2) containing 0.5M sorbitol (washing buffer) and treated at 4 °C for 30min with 1% Triton X-100 (1% final concentration) using a detergent:protein ratio of 8. The solution was then brought to a final concentration of 48% (w/w) sucrose, overlaid with 2ml of 40, 35, 30, and 5% (w/w) sucrose in washing buffer, and centrifuged for 16h at 150000g at 4 °C.

Nine fractions of equal volume were collected from the top to the bottom of the gradient. Proteins corresponding to half the volume of each fraction were precipitated in 10% cold trichloroacetic acid for 30min at 4 °C. After centrifugation, the pellets were washed with 10% trichloroacetic acid in water to remove residual sucrose and then with cold acetone before being resuspended in Laemmli buffer for SDS-PAGE. Precipitated proteins were analysed by western blotting with antibodies to remorin (antibodies kindly provided by S. Mongrand) and to GFP (Invitrogen) for revealing GFP-Phyl1.1. The other half of the volume of each fraction was used to determine the total amount of proteins by the bicinchoninic acid protein assay to avoid Triton X-100 interference, using BSA as a protein standard.

### Structural modelling

The protein regions corresponding to the LDs of the four Arabidopsis phytolongins were determined according to Vedovato *et al.* (2009) and used as target sequences for creating structural models via the Phyre2 server (Kelley *et al.*, 2015), using the VAMP7 LD as a template. Then, the four models were viewed to depict both surface and cartoon (via partial transparency) representations via Pymol (Seeliger and de Groot, 2010), which was also used to generate a movie of the Phyl1.1 LD.

## Results

### Subcellular localization of phytolongins

**Fig. 1.**
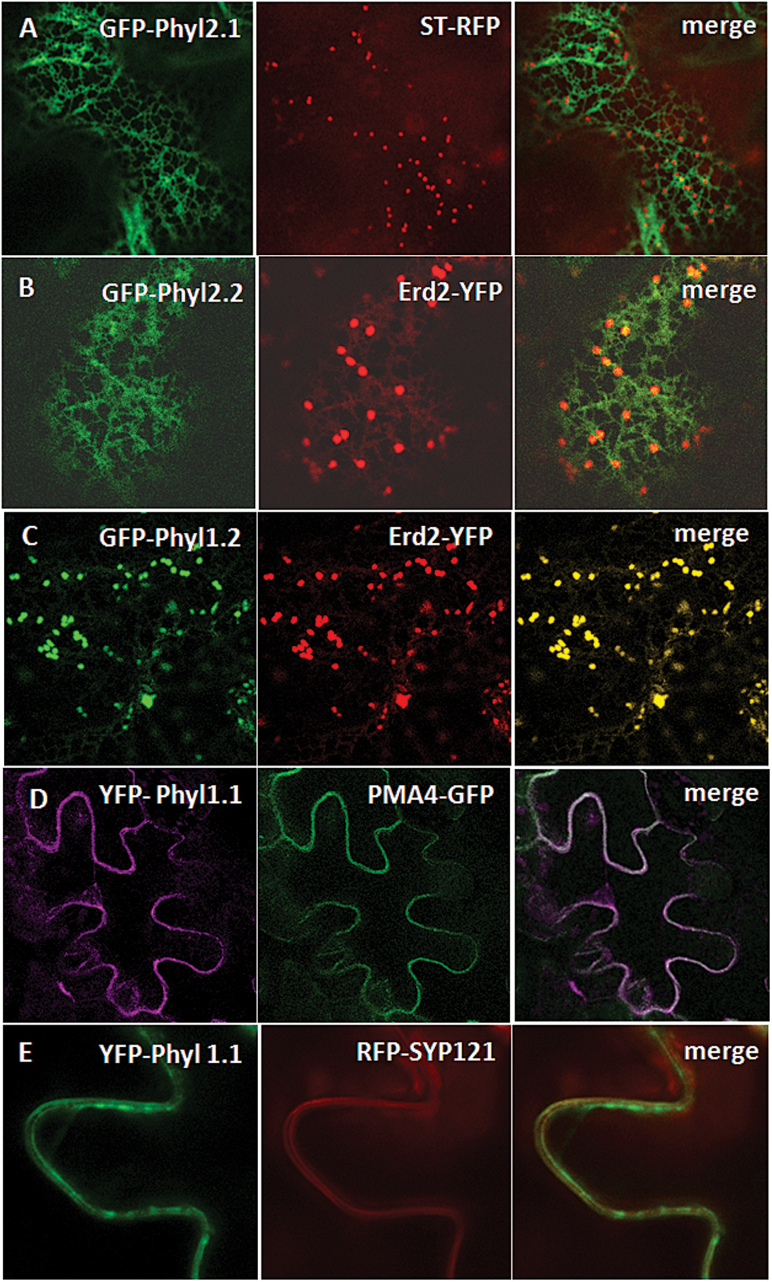
Subcellular localization of the four phytolongins in tobacco leaf epidermal cells. (A, B) GFP-Phyl2.1 and GFP-Phyl2.2 appear to be localized in the ER network. (C) GFP-Phyl1.2 shows a typical co-labelling of the Golgi bodies as observed through its co-expression with the Golgi marker Erd2-YFP. (D, E) YFP-Phyl1.1 co-localized with the plasma membrane markers PMA4-YFP (D) and RFP-SYP121 (E).

First, we compared N- and C-terminal tagging of Phyl1.1 and observed that C-terminal tagged Phyl1.1 accumulated in the ER and uncharacterized aggregates close to the ER (not shown), whereas N-terminal tagged Phyl1.1 was able to exit the ER and traffic to the PM (see below; Fig. 1). We therefore decided to use N-terminal tagged Phyl constructs to compare their subcellular localization in *Agrobacterium*-transformed tobacco leaf epidermal cells.

The subcellular localizations of Phyl1.2, Phyl2.1, and Phyl2.2 coupled to GFP at their N-termini were investigated by co-expression with the ER marker RFP-HDEL and Golgi markers Erd2-YFP or ST-RFP (Chatre *et al.*, 2005; Marais *et al.*, 2015; Tolley *et al.*, 2010). GFP-Phyl2.1 and GFP-Phyl2.2 were clearly located in a typical ER network (Fig. 1A, B), and this ER localization was confirmed by co-labelling with the ER marker RFP-HDEL (Supplementary Fig. S1A at *JXB* online). No co-localization was observed for GFP-Phyl2.1 and GFP-Phyl2.2 with the Golgi markers Erd2-YFP or ST-RFP, establishing that these two proteins are exclusively associated with the ER network (Fig. 1A, B). In contrast, GFP-Phyl1.2 was found to be associated with intracellular dots labelled by Erd2-YFP (Fig. 1C), while no co-labelling was detected with the ER network. Thus, we concluded that GFP-Phyl1.2 is restricted to the Golgi complex.

**Fig. 2.**
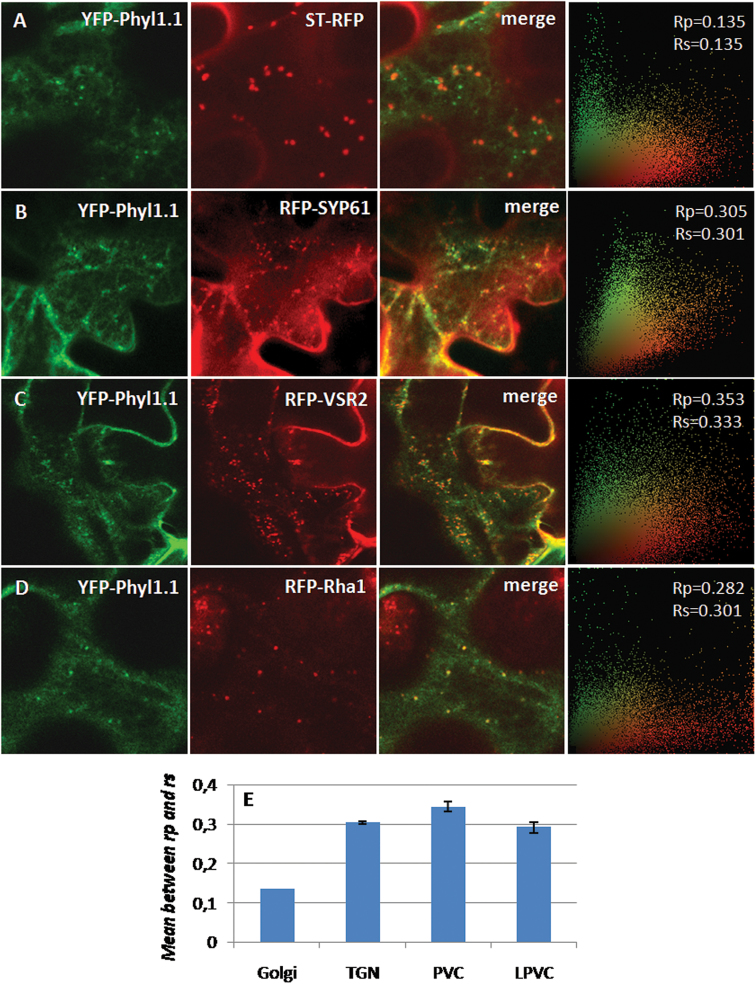
Intracellular localization of the phytolongin Phyl1.1in tobacco leaf epidermal cells. (A, B) YFP-Phyl1.1 co-localized to some extent with the Golgi marker ST-RFP and the TGN marker RFP-SYP61. (C, D) YFP-Phyl1.1 also co-localized to some extent with the PVC marker RFP-VSR2 and the LPVC marker RFP-Rha1. E, Quantification of the co-localization of Phyl1.1 with the various markers, determined according to Gershlick *et al.* (2014). The far right panels in A–D show scatter plots derived from the correlation analysis.

Phyl1.1 coupled to YFP at its N-terminus was mainly located at the PM, as shown by co-labelling with the PM markers PMA4-GFP (Melser *et al.*, 2010) (Fig. 1D) and RFP-SYP121 (Fig. 1E). Moreover, Phyl1.1 was not associated with the tonoplast, as shown by the absence of co-labelling with the tonoplast marker RFP-CBL6 (Bottanelli *et al.*, 2011) (Supplementary Fig. S1B). Phyl1.1 was also found to partially co-localize with the Golgi marker ST-RFP (Fig. 2A), the *trans*-Golgi network (TGN) marker RFP-SYP61 (Bottanelli *et al.*, 2012; Foresti *et al.*, 2010) (Fig. 2B), the prevacuolar compartment (PVC) marker RFP-VSR2 (Bottanelli *et al.*, 2011, 2012; Foresti *et al.*, 2010; Gershlick *et al.*, 2014) (Fig. 2C), and the late prevacuolar compartment (LPVC) marker RFP-Rha1 (Gershlick *et al.*, 2014) (Fig. 2D). For each pair, the statistical level of co-localization is represented in Fig. 2E (Foresti *et al.*, 2010) and shows an equal distribution across the post-Golgi compartments (TGN, PVC, and LPVC). Hence, Phyl1.1 is mainly located at the PM, with some minor accumulation at post-Golgi compartments.

**Fig. 3.**
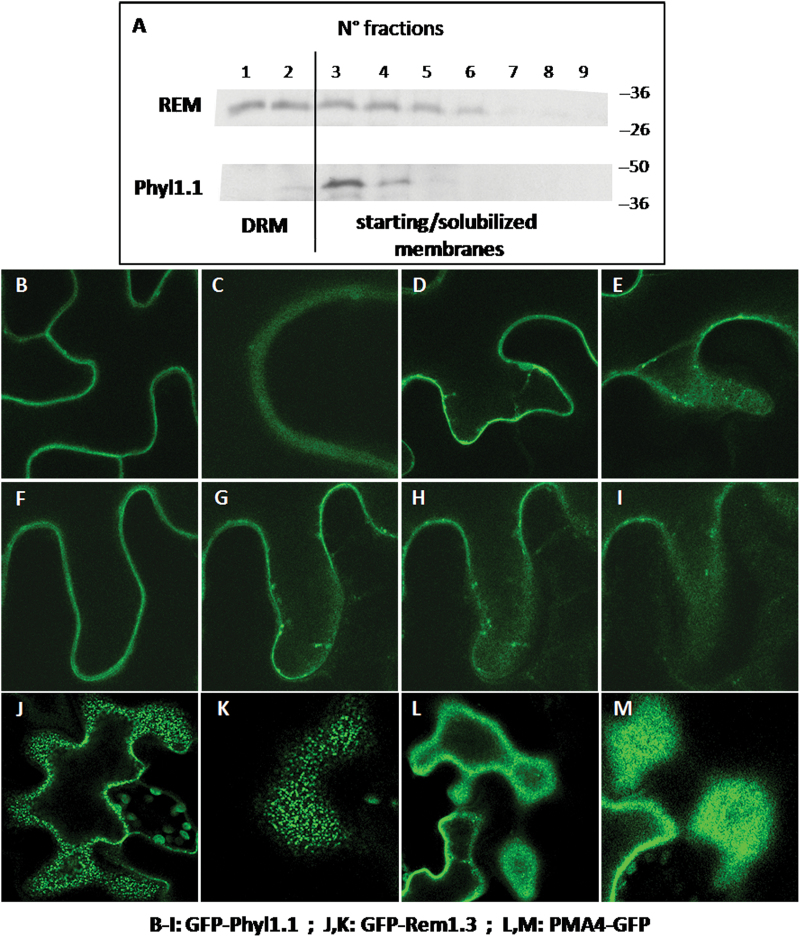
Phyl1.1 is not recovered in DIM-enriched fractions and does not appear in the same pattern as remorin-like domains *in situ*. (A) Remorin (REM; a DIM marker) is enriched in fractions 1 and 2 (DIM, <10% of total proteins) and present in starting/solubilized membranes (fractions 3–9, >90% of total proteins). However, Phyl1.1 is not enriched in the DIM fractions and is mostly found in the starting/solubilized membranes. GFP-Phyl1.1-transformed leaves of 4-week-old tobacco plants were homogenized and centrifuged to produce a microsomal pellet. Triton X-100 lysis of membranes, separation into nine fractions on a sucrose gradient, and protein analysis by western blotting with antibodies to remorin and to GFP for revealing GFP-Phyl1.1 were performed as described in the Materials and methods. (B–M) When GFP-Phyl1.1 at the plasma membrane is analysed at the tangential plane by confocal microscopy (B–I), it behaves in exactly the same way as PMA4-GFP (L, M), which is not recovered in DIM, but not in the same way as GFP-Rem1.3 (J, K), which is a marker of DIM. (F–I) Images show slices from a z-stack.

As it is known that PM is a heterogeneous membrane displaying lateral segregation in membrane microdomains rich in sterols that can influence SNARE activity, we investigated whether the non-SNARE Phyl1.1 could be associated with such a sterol-rich environment. To assess this, we tested the potential presence of Phyl1.1 in detergent-insoluble membranes (DIM) at the PM, which is known to be enriched in sterols. Tobacco leaf epidermal cells were infiltrated with YFP-Phyl1.1, and microsomal membranes were prepared from infiltrated leaves and treated with Triton X-100 to separate the DIM from the solubilized membranes (Laloi *et al.*, 2007; Raffaele *et al.*, 2009). As shown in Fig. 3A, Phyl1.1 does not appear in the same pattern of membrane fractions as the DIM marker remorin (Raffaele *et al.*, 2009), and therefore is probably not associated with the corresponding domains of the PM. This result was confirmed by analysing GFP-Phyl1.1 at the PM in the tangential plane by confocal microscopy (Fig. 3). Effectively, GFP-Phyl1.1 (Fig. 3B–I) behaves similarly to PMA4-GFP (Fig. 3L, M), which is not recovered in DIM, and unlike GFP-Rem1.3 (Fig. 3J, K), which is recovered in and is a marker of DIM (Perraki *et al.*, 2012; Raffaele *et al.*, 2009). The few dots observed for GFP-Phyl1.1 (Fig. 3B–I) may correspond, at least in part, to Golgi structures or PVC as revealed using Golgi and PVC markers (Fig. 2).

On the basis of these results, it appears that the four phytolongins are distributed along the secretory pathway, with Phyl2.1 and Phyl2.2 localized only in the ER network, Phyl1.2 associated with the Golgi apparatus, and Phyl1.1 having a major localization at the PM but also being present to different extents in the Golgi, TGN, PVC, and LPVC. In addition, Phyl1.1 does not segregate into PM subdomains such as those labelled by the ‘raft’ marker remorin (Perraki *et al.*, 2012; Raffaele *et al.*, 2009).

### Phyl1.1 follows the secretory pathway to reach the PM

**Fig. 4.**
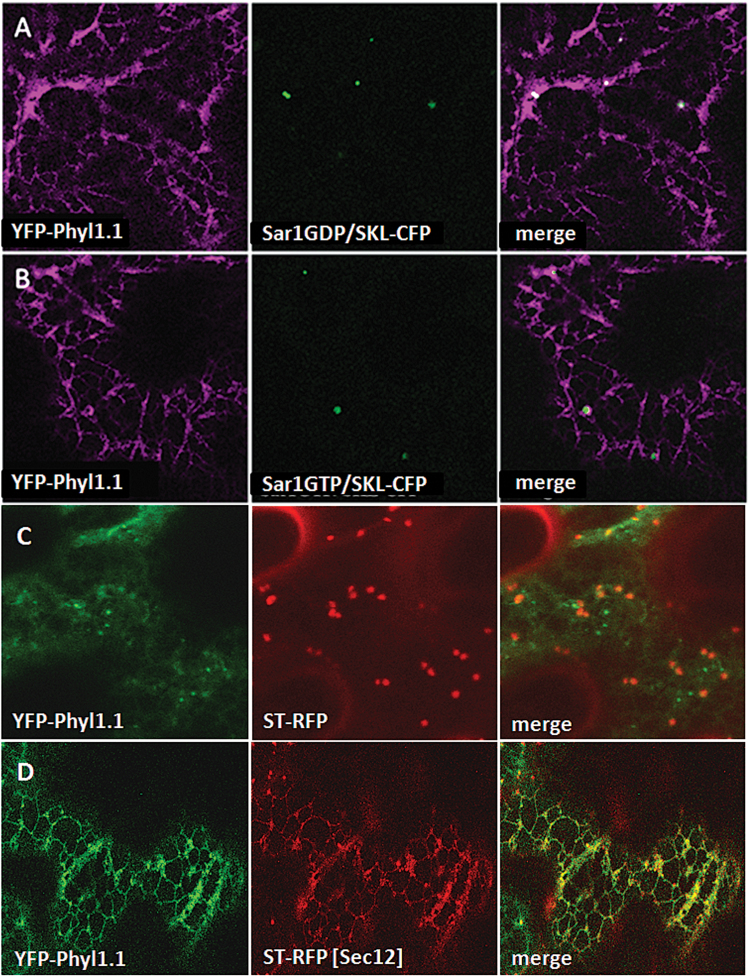
Over-expression of Sar1 mutants and of Sec12 induces the redistribution of YFP-Phyl1.1 to the ER. (A, B) Expression of the mutant blocked forms of Sar1, Sar1-GDP and Sar1-GTP, inhibits transport of the protein YFP-Phyl1.1 to the PM and maintains it in the ER network. (C, D) Over-expression of Sec12 redistributes YFP-Phyl1.1 together with the co-expressed ST-RFP into the ER (D) compared with the control (C).

In a first attempt to determine whether Phyl1.1 follows the secretory pathway to reach the PM, we over-expressed the GDP- and GTP-blocked mutant forms of Sar1 together with YFP-Phyl1.1. The mutant forms of Sar1 are known to be able to affect ER–Golgi transport (Hanton *et al.*, 2008; Takeuchi *et al.*, 2000) and therefore to induce the retention of cargoes at the level of the ER membranes. Co-expression of SKL-CFP allowed screening for the fluorescent cells where these mutant forms of Sar1 were effectively expressed. YFP-Phyl1.1 was effectively retained in the ER network when either GDP- or GTP-blocked mutant forms of Sar1 were over-expressed (Fig. 4A, B). In addition, Phyl1.1 export from the ER was inhibited by co-expression of Sec12 (Fig. 4C, D). The over-expression of Sec12, which disturbs ER–Golgi traffic, was guaranteed by the co-expression of the Golgi marker ST-RFP on a dual vector (within the same T-DNA; Bottanelli *et al.*, 2011). YFP-Phyl1.1, together with the ST-RFP Golgi marker, was partly trapped in the ER network when Sec12 was over-expressed.

**Fig. 5.**
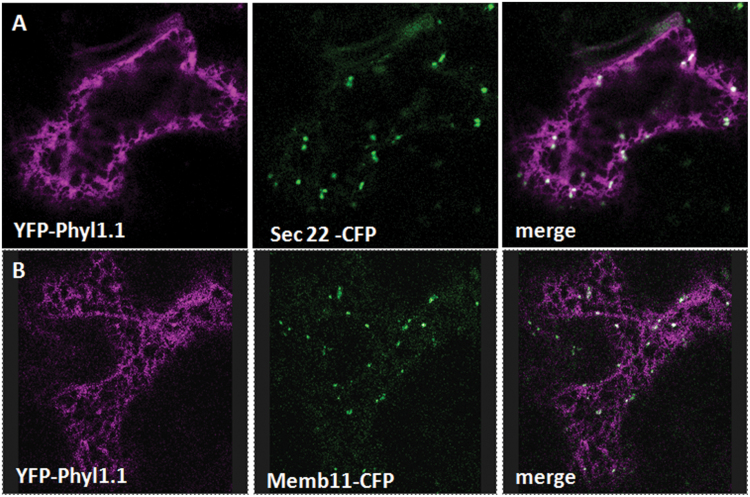
Over-expression of the SNAREs Sec22-CFP (A) and Memb11-CFP (B) redistributes YFP-Phyl1.1 from the PM into the ER.

To confirm the results obtained with the over-expression of the GDP- and GTP-blocked mutant forms of Sar1 and the over-expression of Sec12, we also over-expressed the fusion constructs Sec22-CFP and Memb11-CFP together with YFP-Phyl1.1, since it has been shown that over-expression of the SNAREs Sec22 and Memb11 can block soluble and membrane-associated cargoes in the ER (Chatre *et al.*, 2005; Bubeck *et al.*, 2008). Indeed, we found that blocking the early secretory pathway at the ER–Golgi step by over-expressing Sec22-CFP (Fig. 5A) or Memb11-CFP (Fig. 5B) retained YFP-Phyl1.1 in the ER network, strongly suggesting that Phyl1.1 is effectively transported to the PM through the secretory pathway.

### Trafficking motifs of Phyl1.1 and Phyl1.2

The differential localization of Phyl2 proteins at the ER and Phyl1 proteins at the Golgi/PM led us to investigate whether a specific motif/sequence in Phyl1 proteins was responsible for exit from the ER. Phytolongins are single-pass type IV proteins anchored to the membrane via their C-terminal TMD anchor (Borgese *et al.*, 2003; Burri and Lithgow, 2004; Chou and Shen, 2007). Since TMD length has been shown to influence the targeting of a membrane protein (Brandizzi *et al.*, 2002a), we decided to shorten the TMD by deletion of the residues highlighted in red in Fig. 7. Our results show that shortening the TMD by six amino acids did not influence the transport of YFP-Phyl1.1 to the PM (mutant YFP-Phyl1.1 *TMD*; Fig. 7C). We then searched for motifs potentially involved in ER sorting and found two YF motifs as potential ER export signals (Barlowe, 2003; Matheson *et al.*, 2006) in the amino acid sequence of Phyl1.1.

**Fig. 6.**
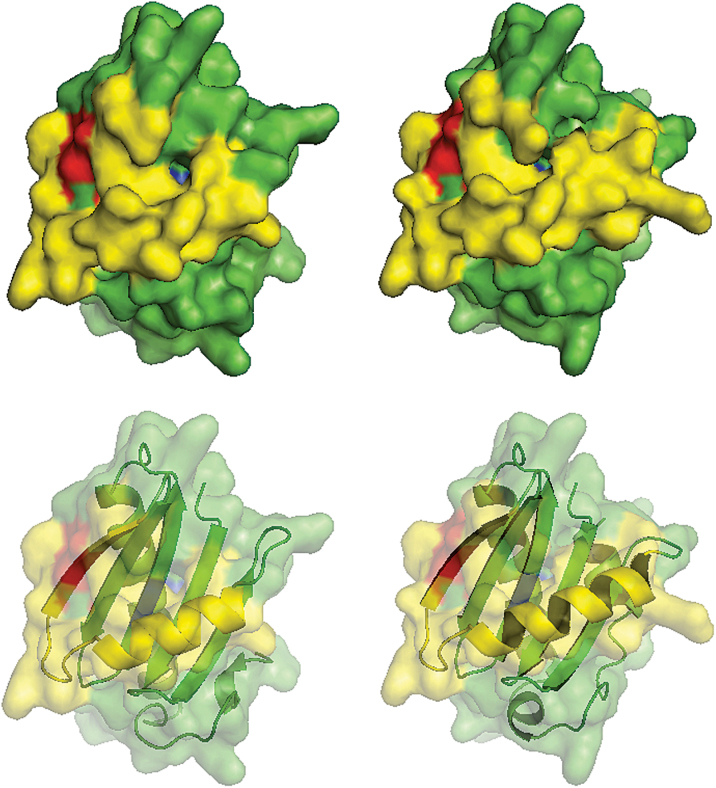
Structural models for the Phyl1.1 and Phyl 1.2 longin domains. Opaque (top) and partially transparent (bottom, to highlight secondary structure) surface representations of the structural models for the LD of Phyl1.1 (left images) and Phyl1.2 (right images). The α1-β3 region is highlighted in yellow. The YF motif specific to Phyl1.1 and Phyl1.2 (red) is surface exposed, while the YF motif shared by all four phytolongins (blue) is buried in the LD core.

In order to investigate conservation and obtain predictive indications of the possible role of the two motifs, a secondary structure-based alignment of phytolongins was performed (Supplementary Fig. S2). The Y67F68 motif is shared by all four Arabidopsis phytolongins, but these show different subcellular locations, and thus the motif is unlikely to be involved in targeting specificity. However, the Y48F49 motif is shared by Phyl1.1 (PM localization) and Phyl1.2 (Golgi localization) but is absent from Phyl2.1 and Phyl2.2 (ER localization). Therefore, the Y48F49 motif might be involved in mediating exit from the ER, allowing for the differential subcellular localizations among the two Phyl1 and the two Phyl2 proteins. Sorting signals are able to influence protein interactions and thus subcellular sorting, if such motifs are surface exposed. Therefore, to investigate the three-dimensional positioning of the two YF motifs (Y48F49 versus Y67F68), structural models of the LDs of the four phytolongins were created. Fig. 6 shows that in both Phyl1.1 and Phyl1.2 LDs, the Y48F49 motif (red) is solvent exposed, as it belongs to the β3 strand, which in turn is part of the α1-β3 region (yellow) known to play an important role in LD regulation and subcellular targeting in all other longins (Mancias and Goldberg, 2007; Pryor *et al.*, 2008; Tochio *et al*, 2001; Vivona *et al.*, 2010; Wen *et al.*, 2010). Instead, the Y67F68 motif (blue) is not accessible in all phytolongins, considering that it is part of the β5 strand in the core region of the LD (see Fig. 6 and Supplementary Fig. S3). A complete three-dimensional view of the Phyl1.1 LD showing the two YF motifs from any angle is presented in the Supplementary video S1. To experimentally check the putative role of the surface-exposed motif in the transport of Phyl1.1 to the PM, it was replaced by site-directed mutagenesis by GG (mutations *Y48G/F49G*).

**Fig. 7.**
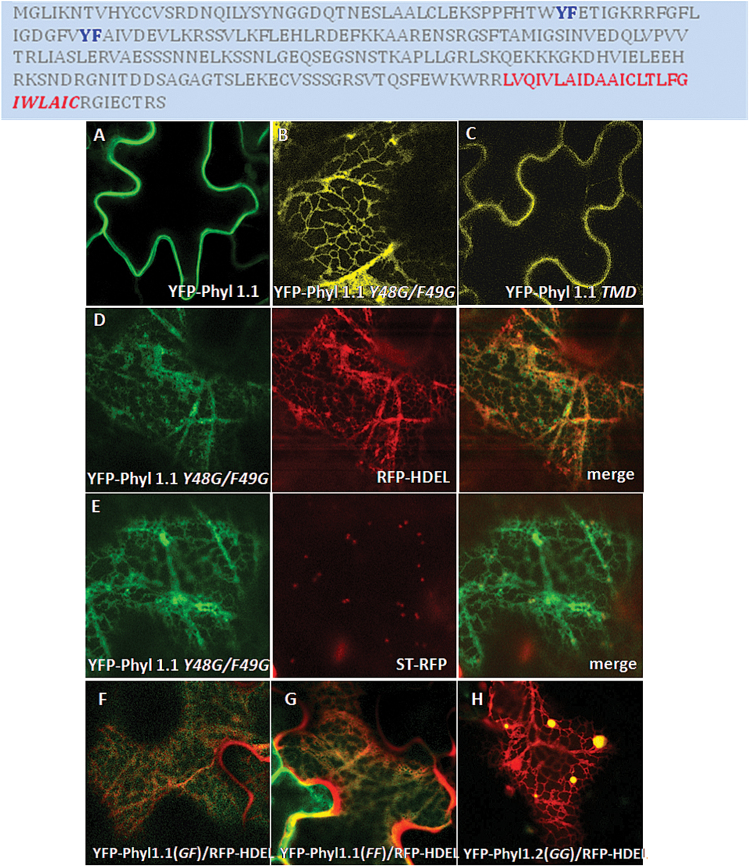
Trafficking motifs of Phyl1.1. Top: Amino acid sequences of Phyl1.1 and its mutated regions. Two putative ER export motifs, YF, are highlighted in blue; the TMD (24 amino acids; highlighted in red) was shortened, deleting the six amino acids in italic text. (A) Normal localization of YFP-Phyl1.1 at the PM. (B) The mutated YFP-Phyl1.1*Y48G/F49G* is retained in the ER network. (C) The mutated YFP-Phyl1.1*TMD* is localized at the PM, indicating that shortening the TMD did not affect its transport to the PM. (D, E) Co-expression of YFP-Phyl1.1*Y48G/F49G* with the ER marker RFP-HDEL and the Golgi marker ST-RFP. (F, G) Retention of YFP-Phyl1.1(*GF*)(*Y48G/F49*) and YFP-Phyl1.1(*FF*)(*Y48F/F49*) at the beginning of the secretory pathway (mostly in the ER) by co-expression with the respective ER marker RFP-HDEL. (H) Phyl1.2(*GG*)(*Y50G/F51G*) forms large aggregates co-localizing with the ER network.

Compared with the PM localization of YFP-Phyl1.1 (Figs 1 and 7A), mutating the putative ER export motif Y48F49 to *Y48G/F49G* led to the retention of YFP-Phyl1.1 in the ER (mutant YFP-Phyl1.1 *Y48G/F49G*, Fig. 7B). Retention of YFP-Phyl1.1 *Y48G/F49G* at the beginning of the secretory pathway (mostly in the ER) was confirmed by the strong co-localization of YFP-Phyl1.1 *Y48G/F49G* with the ER marker RFP-HDEL, while no co-localization was observed with the Golgi marker ST-RFP (Fig. 7D, E).

As tyrosine is largely present in linear motifs, and because tyrosine is often the major determinant (Bonifacino and Dell’Angelica, 1999), the *Y48G/*F49 mutant was performed, and since a mutation of tyrosine by phenylalanine has generally no impact on folding, we also designed the *Y48F/*F49 mutant. The results obtained with the Phyl1.1 *Y48G/*F49 and Phyl1.1 *Y48F/*F49 mutants show that the proteins were retained in the ER (Fig. 7F, G). This is in strong favour of the hydrophobic Y48F49 motif being critical for ER export and also indicates the importance of the tyrosine residue Y48.

Since a similar motif is present in Phyl1.2 (Supplementary Fig. S2), we also designed the Phyl1.2 *Y50G/F51G* mutant and observed the formation of large aggregates co-localizing with the ER (Fig. 7H). Therefore, the di-aromatic amino acid motif YF is clearly an essential ER export signal for Phyl 1.1 and Phyl1.2 trafficking.

## Discussion

The phytolongins are a new set of proteins within the longins family. Interestingly, while the brevin subclass of R-SNAREs diversified in animals, fungi, and yeasts, in plants the longin subclass diversified. Phytolongins do not have a SNARE domain but instead contain another uncharacterized domain, the Phyl domain. When considering the role played by the SNARE domain in subcellular trafficking, its absence in phytolongins might suggest they are not trafficking functional. However, this is contradicted by evidence for the wider role in subcellular trafficking played by the LD, alone or in combination with domains other than SNARE. In fact, up to seven families of proteins endowed with an LD are conserved in eukaryotes and involved in most subcellular trafficking events/complexes; of these families, only the *sensu stricto* longins (prototyped by VAMP7, Sec22, and Ykt6) is also endowed with a SNARE domain (De Franceschi *et al.*, 2014). Therefore, the association between the LD and subcellular trafficking is common, and even those *sensu stricto* longins that are lacking a SNARE domain (e.g., the mammalian Sec22b homologues Sec22a/c) are still involved in subcellular trafficking (Rossi *et al.*, 2004).

Our results show that the four phytolongins Phyl1.1, Phyl1.2, Phyl2.1, and Phyl2.2 are targeted to different membranes of the secretory pathway. While Phyl2.1 and Phyl2.2 are exclusively present in the ER network, Phyl1.2 is associated with the Golgi bodies and Phyl1.1 is mainly localized at the PM but also distributed to some extent in the Golgi, the TGN, the PVC, and the LPVC compartments. Moreover, Phyl1.1 does not segregate into DIM domains and is randomly distributed at the cell surface. This indicates that, in contrast to several SNARE proteins for which localization in membrane domains of the PM enriched in sterols has been shown, Phyl1.1 protein, which does not contain a SNARE domain, does not display this lateral segregation in membrane domains. Differential localization of the Phyl2 and Phyl1 sets of proteins led us to determine how this distinction is made during trafficking. We demonstrated that Phyl1.1 protein traffics through the ER, by over-expression of the GDP- and GTP-blocked mutant forms of Sar1, of Sec12, and of the SNAREs Sec22 and Memb11, which blocked Phyl1.1 at the level of the ER. Moreover, we found evidence that a surface-exposed YF motif present within the α1-β3 region of the LD is critical for the ER export of Phyl1.1 (the Y48F49 motif) and that such a motif (the Y50F51 motif) is potentially also required for the export of Phyl1.2 from the ER. In addition, we also found evidence supporting the critical importance of the tyrosine Y48 of Phyl1.1 for its ER export.

Phytolongins are characterized by the presence of a small hydrophobic site followed C-terminally by several positively charged residues. This PLLG[K/R]--[K/R]--KKK[K/G][K/R] sequence of 15 amino acids is present in all phytolongins so far identified and does not appear in proteins of the VAMP7 family, as shown for VAMP727 (Supplementary Fig. S4); in addition, a hydrophilic loop, similar to that in the SNARE motif of Sec22 (NIE hydrophilic loop) but localized in the Phyl domain, is also present (Vedovato *et al.*, 2009). In the mammalian Sec22 protein, the NIE hydrophilic loop in the SNARE domain interacts with the LD to form an inactive SNARE, which is incapable of forming a SNARE complex due to intramolecular folding masking its SNARE motif (Mancias and Goldberg, 2007). Indeed, binding of the VAMP7 LD to the SNARE motif is crucial to both regulation of membrane fusion (by inhibiting participation in the SNARE bundle) and subcellular localization, as once the SNARE-binding region of the LD is not occupied by the SNARE motif, the LD can interact with other protein complexes, and this in turn governs VAMP7 subcellular sorting (Pryor *et al.*, 2008). The presence of a hydrophilic ‘NIE-like’ loop in the Phyl domain suggests that the LD of phytolongins could also fold in a so-called ‘closed’ conformation. This is further suggested by evidence that (i) in VAMP7 only the N-terminal part of the SNARE motif is crucial to, and sufficient for, mediating intramolecular binding to the SNARE motif (Vivona *et al.*, 2010), and (ii) when aligning plant VAMP7 SNARE motifs with phytolongin Phyl regions, conservation of both the heptadic hydrophobic layers and of the ‘NIE-like’ region is observed in the N-terminal half, while conservation is lost in the C-terminal half (Vedovato *et al.*, 2009; Fig. 5), which in VAMP7 is instead crucial to SNARE bundle formation (Vivona *et al.*, 2010). In practice, the N-terminal halves of both the Phyl regions and the VAMP7 SNARE motifs might share the conserved capacity to regulate LD binding to other complexes, hence subcellular sorting, while SNARE motifs and Phyl regions would diverge in their capacity to participate, or not, in SNARE bundles. Our data on the Y48F49 motif fit with such a hypothesis. In agreement with this motif being involved in Phyl1.1 and Phyl1.2 subcellular sorting, it is located in the α1-β3 region, which is predicted to mediate intramolecular LD binding to the N-terminus of the Phyl region (Vedovato *et al.*, 2009), and thus, in turn, to regulate LD interactions crucial to determine subcellular sorting, as reported for Sec22b and VAMP7 (Mancias and Goldberg, 2007; Pryor *et al.*, 2008). In any case, the lack of a standard SNARE motif in phytolongins leaves open the possibility that the ‘NIE-like’ loop of the phytolongins could also interact with the LD of SNAREs (VAMP7, YKT6, Sec22, etc.) to regulate their availability to form SNARE complexes, this activity being itself potentially regulated by the interaction of the hydrophilic loop of the phytolongins with their own LD. In addition, phytolongins may also interact with/regulate other LD-containing proteins linked to the function of the secretory pathway, such as some coat subunits, adaptor proteins, sedlins in TRAPP complexes, Rab GTPase exchange factors (GEF) and GEF multi-protein complexes, and the signal recognition particle receptor (De Franceschi *et al.*, 2014; Daste *et al.*, 2015). Microarrays/RNA-seq data analysis with CSB.DB (http://csbdb.mpimp-golm.mpg.de), KOBAS (http://kobas.cbi.pku.edu.cn), GeneMANIA (http://genemania.org/) and Genevestigator (http://genevestigator.com/gv/) indicate that the expression patterns of the phytolongins may follow those of several VAMP7 proteins, as well as YKT62 for Phyl1.2. YKT62 has been shown to interact with SYP41 (Chen *et al.*, 2005), which is located at the Golgi, and Phyl1.2 was found to be located in this subcellular compartment. These features could be compatible with a role of phytolongins in regulating the functionality of SNAREs belonging to the longins family in the secretory pathway of *A. thaliana*. Moreover, the microarrays/RNA-seq data also highlight potential links between Phyl1.1 and several cargo proteins, such as PM intrinsic proteins (PIP), aquaporin-like superfamily proteins, one tonoplast intrinsic protein (TIP), and other proteins of the transport machinery, such as the vacuolar protein sorting receptor VPS55 and Ypt/Rab-GAP domains of Gyp1 family proteins. These potential links are compatible with the observed multi-subcellular localization of Phyl1.1.

More generally, the bioinformatic data point to a potential involvement of the phytolongins in metabolism homeostasis and/or cargo transport for rapid cell growth in relation to germination and/or stress-related events (wound response, hypoxia, drought resistance, etc.).

Looking at the role of Phyl2 proteins at the ER, it must also be noted that, according to GeneMANIA, Phyl2.1 and Phyl2.2 have a similar expression pattern to that of the reticulon-like protein RTNLB11. Reticulon proteins are involved in the regulation of the ER network structure and dynamics (Lee *et al.*, 2013; Sparkes *et al.*, 2010, 2011; Tolley *et al.*, 2008, 2010). Additionally, some reticulon proteins have been found at the PM and have been reported to be involved in regulating the connections between ER and PM in plants (Kriechbaumer *et al.*, 2015). In mammalian and yeast cells, Sec22 (another longin-containing protein localized in the ER) and some PM syntaxins have been implicated in these ER–PM interactions (Daste *et al.*, 2015). Taken together, this opens the possibility that Phyl2 not only acts at the ER level but might be involved at other levels, such as the ER–PM interaction. Future work should address whether Phyl2 proteins can act together with reticulon proteins to regulate ER shaping/dynamics as well as ER–PM interactions, and/or whether they may even have a broader involvement in ER–Golgi transport.

In conclusion, the localization of phytolongins in the plant secretory pathway and the bioinformatic data together open an exciting area of research towards understanding the function of these particular proteins that contain both an LD domain and a new uncharacterized Phyl domain in place of the SNARE domain.

## Supplementary data

Figure S1. Co-localization of YFP-Phyl2.1 with the ER marker RFP-HDEL (A) and the absence of co-localization of YFP-Phyl1.1 with the tonoplast marker RFP-CBL6 (B).

Figure S2. A secondary structure-based alignment of the phytolongins from Arabidopsis and other plant species.

Figure S3. Structural models for the Phyl2.1 and Phyl2.2 longin domains.

Figure S4. A conserved PLLG[K/R]--[K/R]--KKK[K/G][K/R] motif in the sequences of the four phytolongins.

Video S1. A complete three-dimensional view of the structural model of the Phyl1.1 longin domain.

Supplementary Data
