# Supplementary material for: Subcellular localization and trafficking of phytolongins (non-SNARE longins) in the plant secretory pathway
Source: J Exp Bot. 2016 Mar 9;67(9):2627–39. doi: 10.1093/jxb/erw094 (PMC4861013; doi:10.1093/jxb/erw094)
Supplement: Supplementary Data [file supp_67_9_2627__index.html]

Subcellular localization and trafficking of phytolongins (non-SNARE longins) in the plant secretory pathway — Subcellular localization and trafficking of phytolongins (non-SNARE longins) in the plant secretory pathway — Supplementary Data 

# Subcellular localization and trafficking of phytolongins (non-SNARE longins) in the plant secretory pathway

## Supplementary Data

Data files

- supplementary\_figures\_S1\_S4.pdf - Supplementary Data
- supplementary\_movie\_S1.avi - Supplementary Data
