# Supplementary material for: Subcellular localization and trafficking of phytolongins (non-SNARE longins) in the plant secretory pathway
Source: J Exp Bot. 2016 Mar 9;67(9):2627–39. doi: 10.1093/jxb/erw094 (PMC4861013; doi:10.1093/jxb/erw094)
Supplement: Supplementary Data [file supp_erw094_supplementary_figures_S1_S4.pdf]

Supplementary data

Figure S1: Co-expression of Phyl2.1 with RFP-HDEL and Phyl1.1 with RFP-CBL6.
A, GFP-Phyl2.1 is found co-localized with RFP-HDEL in the ER network. B, YFP-Phyl1.1 did not co-localize with the tonoplast marker RFP-CBL6.

Figure S2: Multiple alignment of phytolongin sequences showing the secondary structure of the Longin Domains (LD) and the YF motifs.
The secondary structure of part of the LD is reported by yellow arrows (β sheets) and red cylinders (α-helices).
We report in grey a positive charged residue conserved in all phytolongins but also in all longins. We framed under rectangles the two regions where are present the YF motifs at positions 48-49 and 67-68, and have highlighted in blue those residues that permit a further distinction between the phytolongins Phyl1.1/Phyl1.2 and Phyl2.1/Phyl2.2.

Figure S3: Structural models for the Phyl2.1 and Phyl2.2 Longin Domains (LD).
Opaque (top) and partially transparent (bottom, to highlight secondary structure) surface representations of the structural models for the LD of Phyl2.1 (left images) and Phyl2.2 (right ones). The α1-β3 region is highlighted in yellow. A single YF motif, shared by all four phytolongins (blue), is present and buried in the LD core.

Figure S4: Multiple alignment of phytolongin sequences showing the conserved PLLG[K/R]--[K/R]--KKK[K/G][K/R]motif.
This figure shows the conserved sequence PLLG[K/R]--[K/R]--KKK[K/G][K/R] of 15 aa which is present in the 4 phytolongins (aa 157-171 for Phyl1.1, aa 154-168 for Phyl1.2, aa 169-183 for Phyl2.1 and aa 167-181 for Phyl2.2) which is however absent from the sequence of the proteins of the VAMP7 family as illustrated with VAMP727.

Supplementary movie: Complete 3-dimensional view of the structural model of Phyl1.1 Longin Domain (LD).
The α1-β3 region of the LD is highlighted in yellow and the functionnal ER export motif YF of Phyl1.1 is highlighted in red. This motif is totally surface exposed, while the second YF motif shared by all four phytolongins (blue) is buried in the LD core.

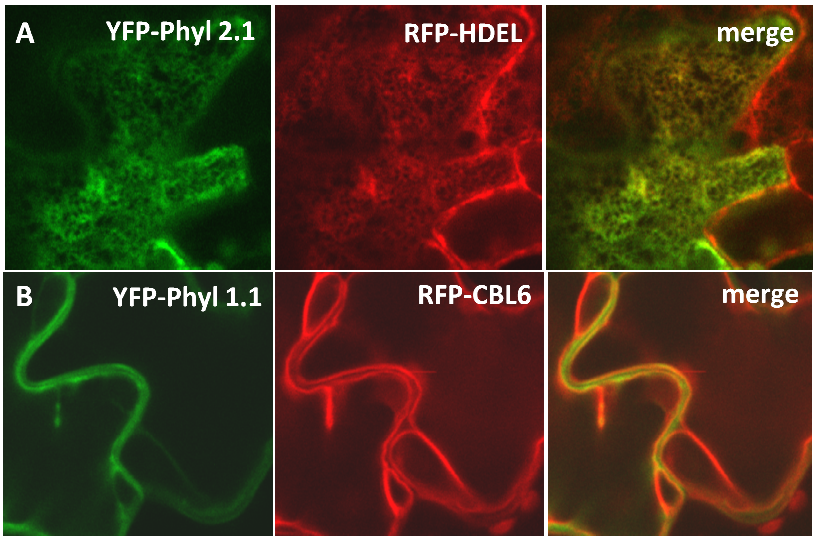

Figure S1

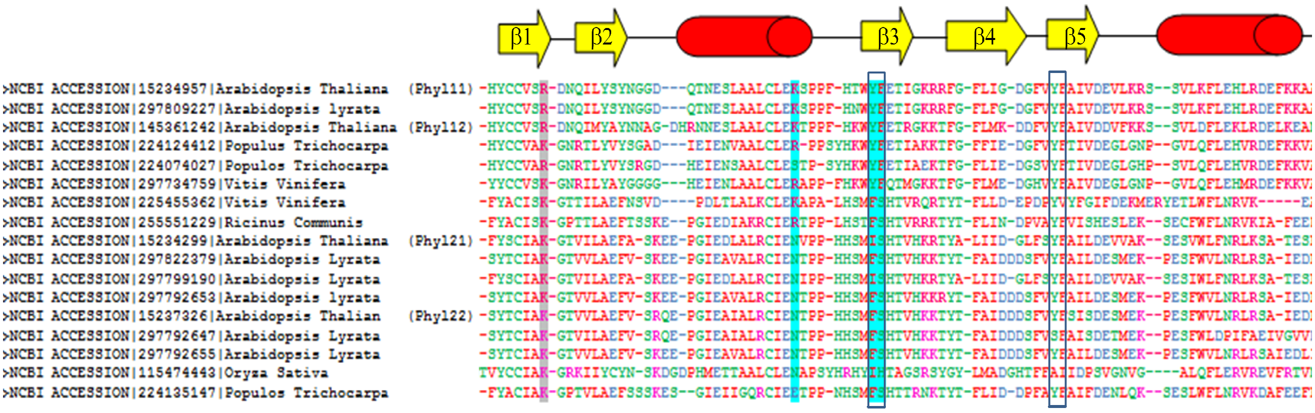

Figure S2

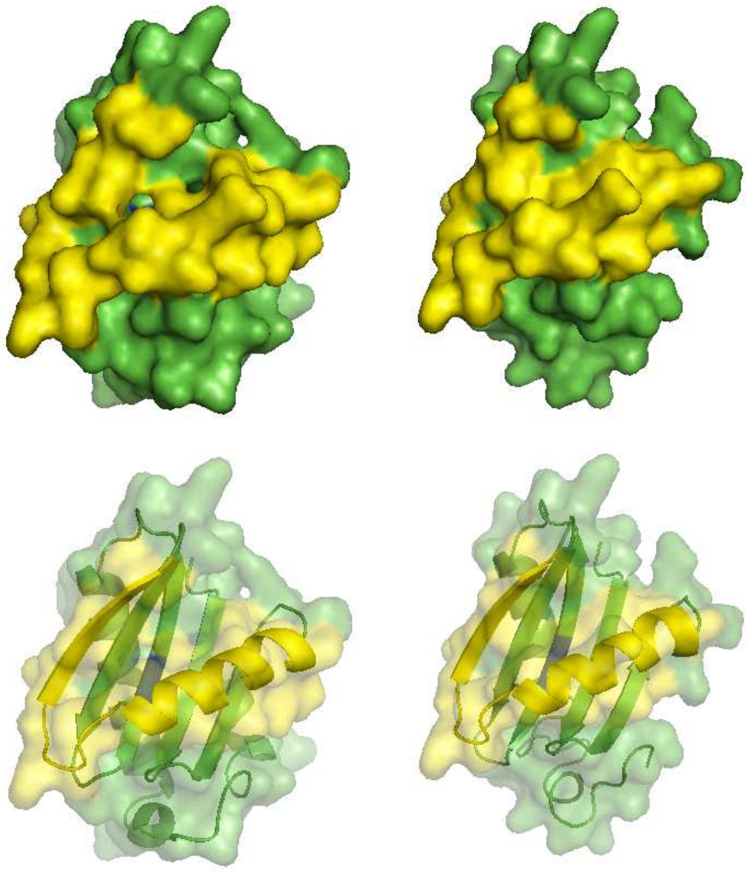

Figure S3

```
Vamp 727   106 ADEDEDDDLFGDRFSVAYNLDREFGPILKEHMQYCMSHPEEMS---------KLSKLKAQ 156
Phyl 1.1   108 GSINVEDQLVPVVTRLIASLERVAESSSNNELKSSNLGEQSE--GSNSTKAPLLGRLSKQ 165
Phyl 1.2   110 SFSNVQDQIV---RRLIASLE-FDHTCLPLSSPSIDGAEQSY--ASNS-KAPLLGRSNKQ 162
Phyl 2.1   118 DPVFAEIAAIGGNHNKDLELEFGSPRSIAREIKSNNQSLDSSKGRKGGALMPLLGKPLRV 177
Phyl 2.2   120 DPVFAEIVGVVDLELLDMDL-VGSPRSVARESR--NPSIDSSKGRR-AALMPLLGKPLKA 175

Vamp 727   157 ITEVKGIMMDN-------IEKVLDRGEKIELLVDKT-----ENLQFQADSFQRQGRQLRR 204
Phyl 1.1   166 EKKKGKDHV-------IELEEHRKSNDRGNITDDSAGAGTSLEKECVSSSGRS-VTQSFE 217
Phyl 1.2   163 DKKKGRDHAHSLRG--IEIEEHRKSNDRGNVTECSN--ASSESATYVPRRGRSGGSQSIE 218
Phyl 2.1   178 LKNKKRLQTEAK--------SEGHWNEKKMDLGGGG------KGVRNGLIHDHHHRQKAK 223
Phyl 2.2   176 LKKKKRLHNEAKGGDSCEVGSIQEISEKNVDLCGNGNNGVLRKELRNGLLSDHHHRQKAK 235
```

Figure S4
